# Evaluating SARC-F, SARC-CalF, and calf circumference as diagnostic tools for sarcopenia in Thai older adults: results from a nationwide study

**DOI:** 10.1186/s12877-024-05637-3

**Published:** 2024-12-28

**Authors:** Ekasame Vanitcharoenkul, Aasis Unnanuntana, Pojchong Chotiyarnwong, Nath Adulkasem, Apichat Asavamongkolkul, Panai Laohaprasitiporn

**Affiliations:** Department of Orthopaedic Surgery, Faculty of Medicine Siriraj Hospital, Mahidol University, 2 Wanglang Road, Bangkok Noi, Bangkok, 10700 Thailand

**Keywords:** Calf circumference, Diagnostic test, Elderly, SARC-F questionnaire, Sarcopenia

## Abstract

**Background:**

With the increasing number of older adults, musculoskeletal disorders such as sarcopenia have become increasingly important to research because of their strong association with falls and fractures. Sarcopenia, which is characterized by reduced muscle mass, is common among older adults and significantly increases the risk of falls. This study aimed to assess the effectiveness of the SARC-F and SARC-CalF questionnaires, along with calf circumference measurements, for sarcopenia screening among Thai community-dwelling older adults, following the 2019 criteria of the Asian Working Group for Sarcopenia.

**Methods:**

This analysis drew on data from the Thai Musculoskeletal Diseases Nationwide Study, which included 2543 participants aged 60 years or older. The SARC-F, SARC-CalF, and calf circumference data were evaluated against the 2019 Asian Working Group for Sarcopenia criteria. We calculated the sensitivity, specificity, and area under the curve to determine the diagnostic performance of each tool.

**Results:**

Of the 2455 participants analyzed, 18.1% were diagnosed with sarcopenia. The SARC-F and SARC-CalF questionnaires showed limited effectiveness in diagnosing sarcopenia, with area under the curve values of 0.508 and 0.729, respectively. In contrast, calf circumference demonstrated greater diagnostic accuracy, with area under the curve values of 0.897 in males and 0.878 in females. Adjusting the cutoff points to < 33 cm for males and < 31 cm for females improved the overall diagnostic accuracy from 66.4 to 82%.

**Conclusions:**

Sarcopenia is relatively prevalent in Thailand. The SARC-F and SARC-CalF questionnaires are inadequate for diagnosing sarcopenia, while calf circumference alone is the most effective screening tool. Adding more parameters to the SARC-F questionnaire could enhance its diagnostic accuracy.

**Trial registration:**

This study was registered at ClinicalTrials.gov (NCT06558617). Registration Date 16 August 2024.

## Background

The growing number of older adults has made diseases affecting the skeletal, joint, and muscular systems increasingly relevant to research. Over the past decade, osteoporosis has gained significant attention because of advancements in antiosteoporotic medications, resulting in a notable decline in fracture rates [1]. However, some patients continue to experience fractures despite appropriate treatment, with falls being a primary contributing factor. Studies indicate that muscle strength plays a crucial role in fall prevention [2]. Sarcopenia, a condition characterized by decreased muscle mass in older adults, contributes significantly to reduced muscle strength and an increased risk of falls [3].

In 2019, the Asian Working Group for Sarcopenia (AWGS) introduced revised diagnostic criteria and updated cutoff values for sarcopenia. These guidelines recommend the SARC-F questionnaire, SARC-CalF questionnaire, and calf circumference as screening tools for detecting sarcopenia in both primary care and clinical research settings [4]. The SARC-F questionnaire has been translated and validated in various languages and clinical contexts [5–9]. Among community-dwelling older adults, the SARC-F and SARC-CalF questionnaires have shown high versatility in identifying sarcopenia cases [10]. The sensitivity of the SARC-F questionnaire ranges from 29.5 to 62.8%, whereas that of the SARC-CalF questionnaire ranges from 56.1 to 83% [11–14]. Calf circumference measurement is a quick, simple method for assessing muscle mass, with multiple studies reporting correlations between calf circumference and both lean mass and physical function [15, 16]. The reported sensitivities and specificities for calf circumference range from 71 to 83.3% and 62.8–84%, respectively [12, 13, 17]. Thus, factors such as sarcopenia prevalence, ethnicity, study population characteristics, and diagnostic criteria may affect the performance of these screening tools.

To our knowledge, no studies have evaluated the performance of these questionnaires and calf circumference measurements for detecting sarcopenia among Thai community-dwelling older adults. This study aimed to assess the effectiveness of these tools in screening for sarcopenia according to the 2019 AWGS criteria through a nationwide cross-sectional analysis in Thailand.

## Methods

### Study design and population

The data for this analysis were derived from the Thai Musculoskeletal Diseases Nationwide Study, a cross-sectional study conducted from March 2021 to August 2022. The study targeted Thai adults aged 60 years and older via a multilevel sampling technique to ensure the representativeness of community-dwelling older adults across 12 provinces, covering Thailand’s six primary geographical regions. The exclusion criteria were individuals with missing data on the SARC-F, SARC-CalF, and calf circumference; those unable to walk independently; bedridden individuals; and those with neuromuscular disorders or severe comorbidities affecting their ability to perform performance-based tests. Six trained research assistants conducted the physical function tests during the study. To ensure consistent instructions, all tests were standardized prior to the study’s commencement.

The study protocol was approved by the Siriraj Institutional Review Board of the Faculty of Medicine Siriraj Hospital, Mahidol University, Bangkok, Thailand (COA-715/2024), and the research was registered with ClinicalTrials.gov (NCT06558617). Trained research assistants obtained written informed consent from all participants before their enrollment.

### Diagnosis of possible Sarcopenia, Dynapenia, and Sarcopenia

To diagnose possible sarcopenia, dynapenia, and sarcopenia, we assessed muscle strength, physical performance, and appendicular skeletal muscle mass (ASM) per the AWGS 2019 criteria. Possible sarcopenia was defined as a decline in muscle strength and/or physical performance. Dynapenia was defined by low muscle function without a decrease in muscle mass [18]. Sarcopenia was characterized by low ASM combined with reduced muscle strength and/or physical performance.

### Muscle strength assessment

Muscle strength was evaluated using handgrip strength measurements. The participants exerted maximum force via a digital Smedley spring hand dynamometer (Takei 5401 Digital Dynamometer; Takei, Tokyo, Japan) while standing with their arms fully extended at their sides. The dynamometer grip size was adjusted for each participant’s hand size, and two to three trials were performed. The highest value recorded was used for analysis. Handgrip strength values of < 28 kg in males and < 18 kg in females were considered indicative of low muscle strength, as per the AWGS 2019 criteria.

### Physical performance evaluation

Physical performance was assessed through gait speed and the five-time sit-to-stand (5TSTS) test. Gait speed was measured over 5 m via a stopwatch, with participants walking at their usual pace along an 11-meter path. The average time to cover the 3- to 8-meter section was calculated. A gait speed of < 1.0 m/s indicated poor performance, per the AWGS 2019 guidelines. The 5TSTS test measures the time required to transition from a sitting position to a standing position and back to sitting; this test is repeated five times. We used a standardized armless chair with a seat height of 43 cm from the floor [19]. The participants sat on a chair with their arms crossed and completed the task as quickly as possible. A duration of ≥ 12 s indicated low physical performance according to the AWGS 2019 criteria.

### ASM measurement

ASM, which represents the combined lean muscle mass of the upper and lower limbs, was measured via bioelectrical impedance analysis (BIA) with a dual-frequency body composition monitor (Tanita RD-545, Tanita Corporation, Tokyo, Japan). The Tanita RD-545 model is recognized for its reliability and validity in determining ASM among older Thai adults [20]. The participants stood barefoot on the metal footpads while holding the device with extended arms. We used height-adjusted ASM values, with cutoffs of < 7.0 kg/m^2^ for men and < 5.7 kg/m^2^ for women, to define low muscle mass according to the AWGS 2019 criteria.

### SARC-F questionnaire

The SARC-F questionnaire includes five components: strength (S), assistance in walking (A), rise from a chair (R), climb stairs (C), and fall (F), reflecting health status changes associated with sarcopenia. Each component is scored from 0 to 2, resulting in a total score ranging from 0 to 10, with scores of ≥ 4 considered abnormal. The Thai version of the SARC-F questionnaire has been validated for use in Thai older adults [5].

### Calf circumference measurement

Calf circumference was measured with nonelastic tape while the participant stood with relaxed legs [21]. To minimize the impact of leg edema on measurement accuracy, measurements were taken in the morning. The measurement was taken at the widest part of the calf, parallel to the floor. An abnormal calf circumference was defined as < 34 cm for males and < 33 cm for females, according to the AWGS 2019 criteria [22].

### SARC-CalF questionnaire

The SARC-CalF integrates the SARC-F score with calf circumference as a trigger for case finding. If the calf circumference was below the cutoff value, 10 points were added to the SARC-F score. The SARC-CalF score ranges from 0 to 20, with a score of ≥ 11 considered abnormal for sarcopenia screening [23].

### Statistical analysis

Continuous variables are reported as means and standard deviations for normally distributed data or medians and interquartile ranges for nonnormally distributed data. The normality of each variable was assessed using the Shapiro–Wilk test. Categorical variables are presented as numbers and percentages. The chi-squared test was used for comparisons between groups. The sensitivity, specificity, positive predictive value, negative predictive value, and area under the receiver operating characteristic curve (AUC) were calculated for the SARC-F, SARC-CalF, and calf circumference, using the AWGS 2019 criteria as the reference standard. The Youden J statistic was used to determine the optimal cutoff point for screening, with an AUC greater than 0.75 indicating clinical relevance by maximizing the sum of sensitivity and specificity [24, 25].

The DeLong and McNemar statistics were used to compare the AUC, sensitivity, specificity, and accuracy between the optimal and AWGS 2019 cutoff values [26, 27]. A *p-*value of < 0.05 was considered to indicate statistical significance. Analyses were performed with IBM SPSS Statistics, version 23 (IBM Corp, Armonk, NY, USA).

## Results

### Study population

**Fig. 1:**
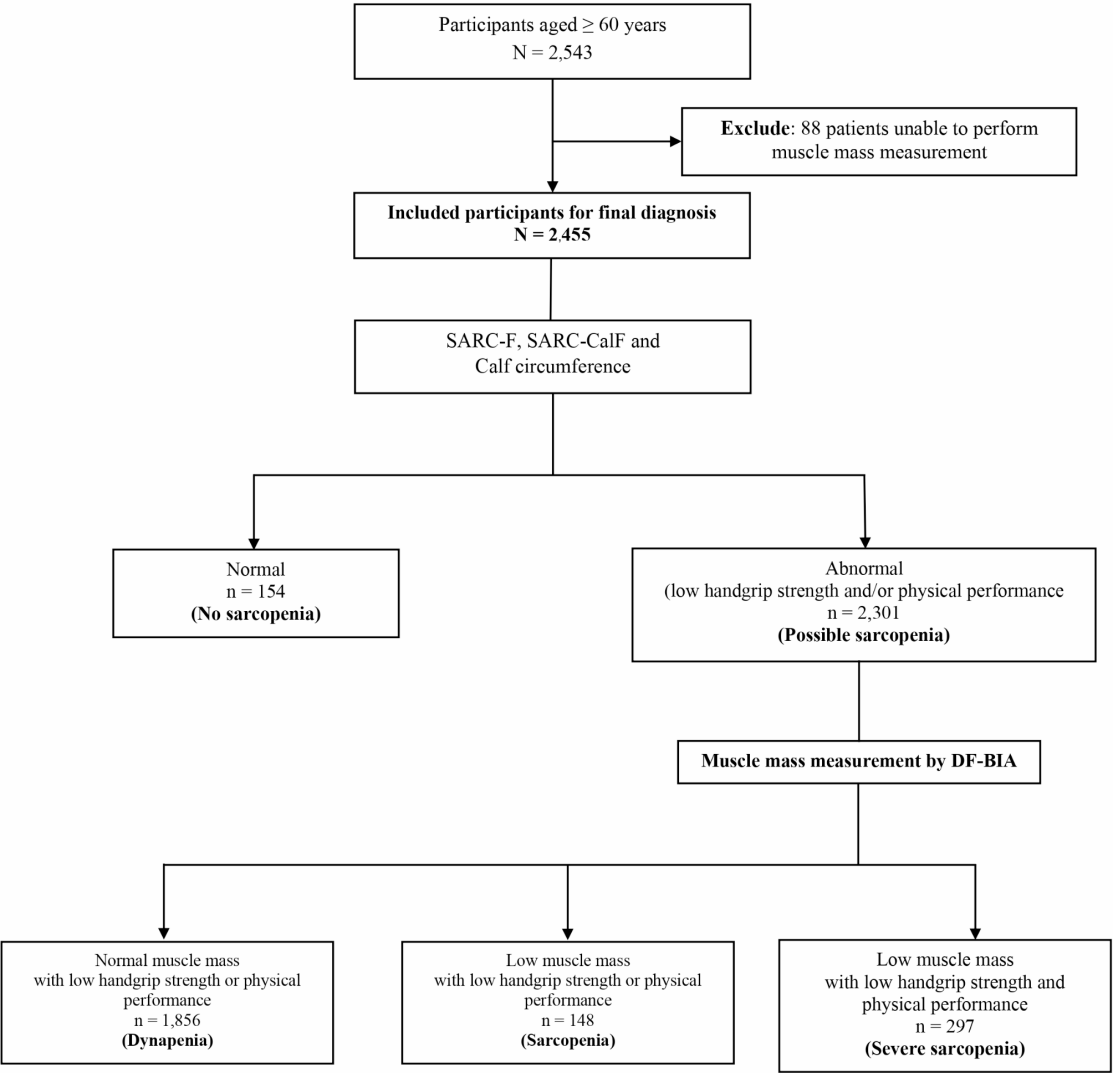
Flowchart of participant selection and diagnostic categorization for sarcopenia screening and diagnosis based on AWGS 2019 criteria; Abbreviations: DF-BIA = dual-frequency bioelectrical impedance analysis

A total of 2543 participants aged 60 years and above were enrolled in the Thai Musculoskeletal Diseases Nationwide Study. Of these, 2455 participants provided complete data for analysis. Among the participants with complete data, 445 were diagnosed with sarcopenia, representing a prevalence of 18.1% **(**Fig. 1**)**. Most participants were female (63.6%), with a mean age of 69 ± 6.1 years.

Statistically significant differences were observed between the SARC-F < 4 and SARC-F ≥ 4 groups in variables such as sex, age, body mass index, Charlson comorbidity index, current smoking history, handgrip strength, ASM, 5TSTS, time-up-and-go test, and fall history. No statistically significant differences were found regarding underlying diseases (chronic obstructive pulmonary disease, diabetes mellitus, or heart failure), history of alcohol use, or percentage of individuals with a suboptimal calf circumference (Table 1).

**Table 1:** Participant demographics data and clinical characteristics

Demographic data and performance tests	Total (*N* = 2455)	SARC-F < 4 (*n* = 2009)	SARC-F ≥4 (*n* = 446)	*p*-value
Female, n (%)	1562 (63.6%)	1185 (59.0%)	377 (84.5%)	< 0.001
Age, year, mean ± SD	69.0 ± 6.1	68.6 ± 5.8	70.7 ± 7.0	< 0.001
Body mass index, kg/m^2^, n (%)				
- < 18.5 - 18.5–24.9 - ≥25.0	197 (8.0%) 1,279 (52.1%) 979 (39.9%)	156 (7.8%) 1,072 (53.4%) 781 (38.9%)	41 (9.2%) 207 (46.4%) 198 (44.4%)	0.029
Charlson comorbidity index, n (%)				
- 1–2 - 3–4 - ≥5	872 (35.5%) 1,368 (55.7%) 215 (8.8%)	757 (37.7%) 1,094 (54.5%) 158 (7.9%)	115 (25.8%) 274 (61.4%) 57 (12.8%)	< 0.001
Chronic obstructive pulmonary disease, n (%)	51 (2.1%)	46 (2.3%)	5 (1.1%)	0.142
Diabetes mellitus, n (%)	438 (17.8%)	347 (17.3%)	91 (20.4%)	0.132
Heart failure, n (%)	19 (0.8%)	14 (0.7%)	5 (1.1%)	0.368
Suboptimal calf circumference, n (%)	1,203 (49.0%)	969 (48.2%)	234 (52.5%)	0.116
Current smoking, n (%)	305 (12.4%)	282 (14.0%)	23 (5.2%)	< 0.001
Alcohol (≥ 3 units/day), n (%)	147 (6.0%)	127 (6.3%)	20 (4.5%)	0.152
Total lean mass, kg, mean ± SD	37.9 ± 7.1	38.6 ± 7.2	34.6 ± 5.5	< 0.001
ASM, kg, mean ± SD	17.3 ± 4.0	17.7 ± 4.1	15.7 ± 3.3	< 0.001
ASM/height^2^, kg/m^2^, mean ± SD	7.1 ± 1.2	7.1 ± 1.2	6.7 ± 1.1	< 0.001
Hand-grip strength, kg, mean ± SD	21.9 ± 7.0	22.9 ± 6.9	17.2 ± 5.0	< 0.001
Gait speed, m/s, mean ± SD	0.9 ± 0.2	0.9 ± 0.2	0.8 ± 0.2	< 0.001
Timed up-and-go test, s, mean ± SD	11.7 ± 3.7	11.0 ± 2.9	14.9 ± 5.1	< 0.001
Five-time sit-to-stand test, s, mean ± SD	16.3 ± 4.8	15.6 ± 4.2	19.8 ± 5.8	< 0.001
History of falling, n (%)	539 (22.0%)	367 (18.3%)	172 (38.6%)	< 0.001

Abbreviations: ASM = appendicular skeletal mass; SD = standard deviation

### Screening for possible sarcopenia

The SARC-F and SARC-CalF tools exhibited high sensitivity rates for screening for possible sarcopenia, with SARC-F reaching 99.8% (95% CI 98.8–99.9) and SARC-CalF achieving 98.8% (95% CI 96.9–99.7). In contrast, the sensitivity of the AWGS 2019 criteria, which are based on calf circumference, was considerably lower at 52.4% (95% CI 48.9–55.9) for males and 47.8% (95% CI 45.3–50.4) for females (Table 2).

**Table 2:** Diagnostic performance of SARC-F, SARC-CalF, and calf circumference for screening possible sarcopenia

Screening tests		AUC (95% CI)	Sensitivity % (95% CI)	Specificity % (95% CI)	PPV % (95% CI)	NPV % (95% CI)
SARC-F (≥ 4)		0.718 (0.684–0.752)	99.8 (98.8–99.9)	7.6 (6.5–8.9)	19.3 (19.1–19.6)	99.4 (95.6–99.9)
AWGS 2019 calf circumference	Male (< 34 cm)	0.528 (0.471–0.585)	52.4 (48.9–55.9)	52.8 (42.0-63.3)	90.7 (88.6–92.5)	11.2 (9.3–13.4)
	Female (< 33 cm)	0.565 (0.503–0.627)	47.8 (45.3–50.4)	63.5 (50.4–75.3)	96.9 (95.7–97.8)	4.9 (4.1–5.8)
SARC-CalF (≥ 11)		0.712 (0.672–0.752)	98.8 (96.9–99.7)	7.1 (6.0-8.2)	14.3 (14.1–14.5)	97.4 (93.3–99.0)
New cut-off calf circumference	Male (< 33 cm)	0.526 (0.463–0.588)	37.5 (34.2–41.0)	68.1 (57.5–77.5)	91.2 (88.4–93.4)	11 (9.6–12.6)
	Female (< 31 cm)	0.557 (0.486–0.628)	30.5 (28.2–32.9)	82.5 (70.9–91.0)	97.7 (96.0-98.6)	4.8 (4.2–5.3)
Revised SARC-CalF (≥ 11)		0.666 (0.620–0.712)	19.3 (17.7–21.0)	93.5 (88.4–96.8)	97.8 (96.1–98.8)	7.2 (6.9–7.5)

Abbreviations: AUC = area under the receiver operating characteristic curve; CI = confidence interval; NPV = negative predictive value; PPV = positive predictive value

**Fig. 2:**
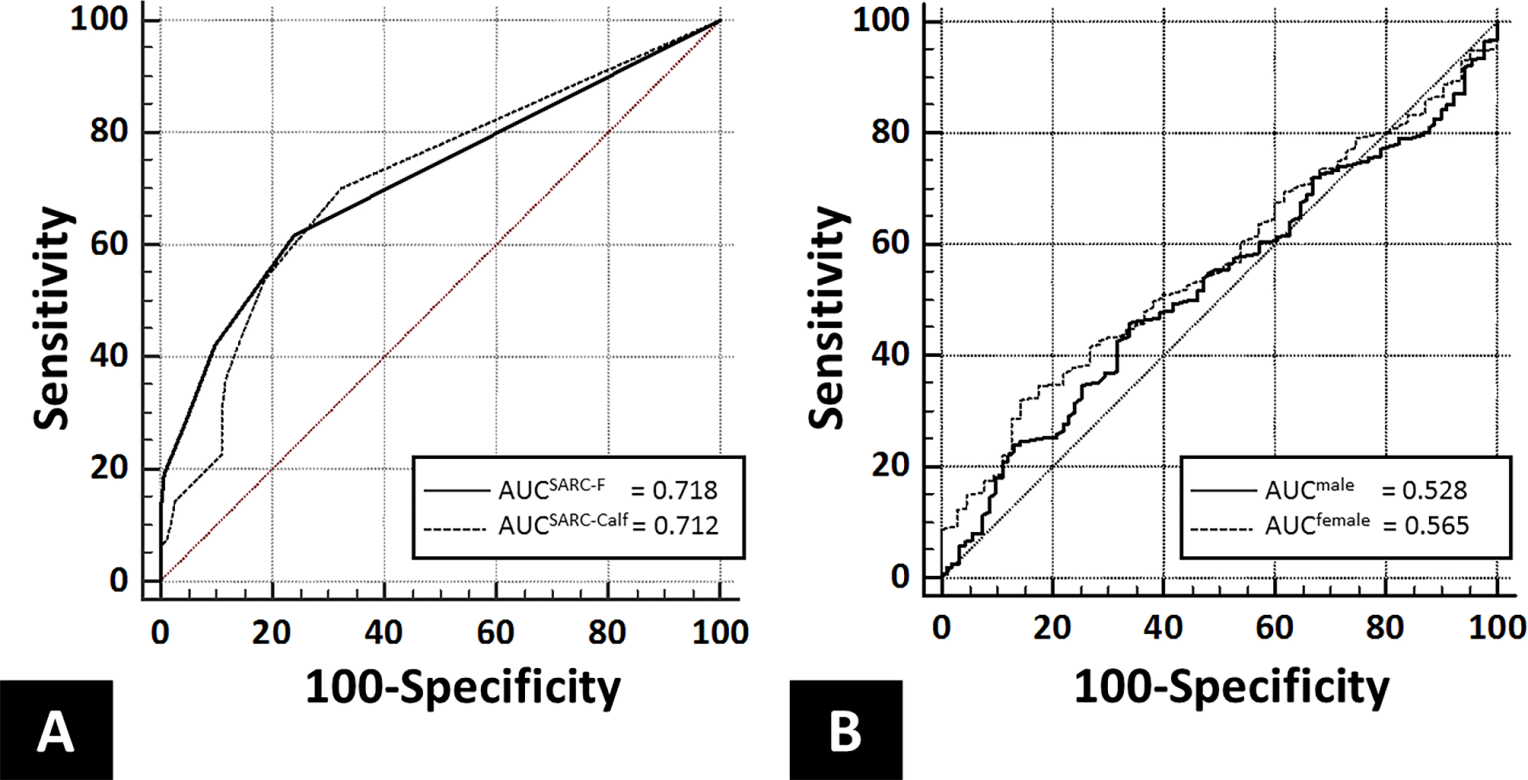
Receiver operating characteristic curves for detecting possible sarcopenia: **(A)** SARC-F and SARC-CalF questionnaires and **(B)** calf circumference measurements

However, the AUC values were 0.718 (95% CI 0.684–0.752) for the SARC-F and 0.712 (95% CI 0.672–0.752) for the SARC-CalF. The AUC values for calf circumference were 0.528 (95% CI 0.471–0.585) in males and 0.565 (95% CI 0.503–0.627) in females. The low AUC values across all three measures indicate limited clinical utility (Fig. 2).

### Effect of new cutoff scores on screening accuracy for possible Sarcopenia

The new cutoff scores derived from our study participants, defined by the maximum Youden index, were applied, and the calf circumference thresholds were set at < 33 cm for males and < 31 cm for females. These revised thresholds were integrated into the SARC-F to evaluate a modified SARC-CalF. However, the AUCs for both the new calf circumference cutoff and the revised SARC-CalF did not indicate a significant increase in screening performance for possible sarcopenia (Table 2).

### Diagnostic performance for Sarcopenia

**Fig. 3:**
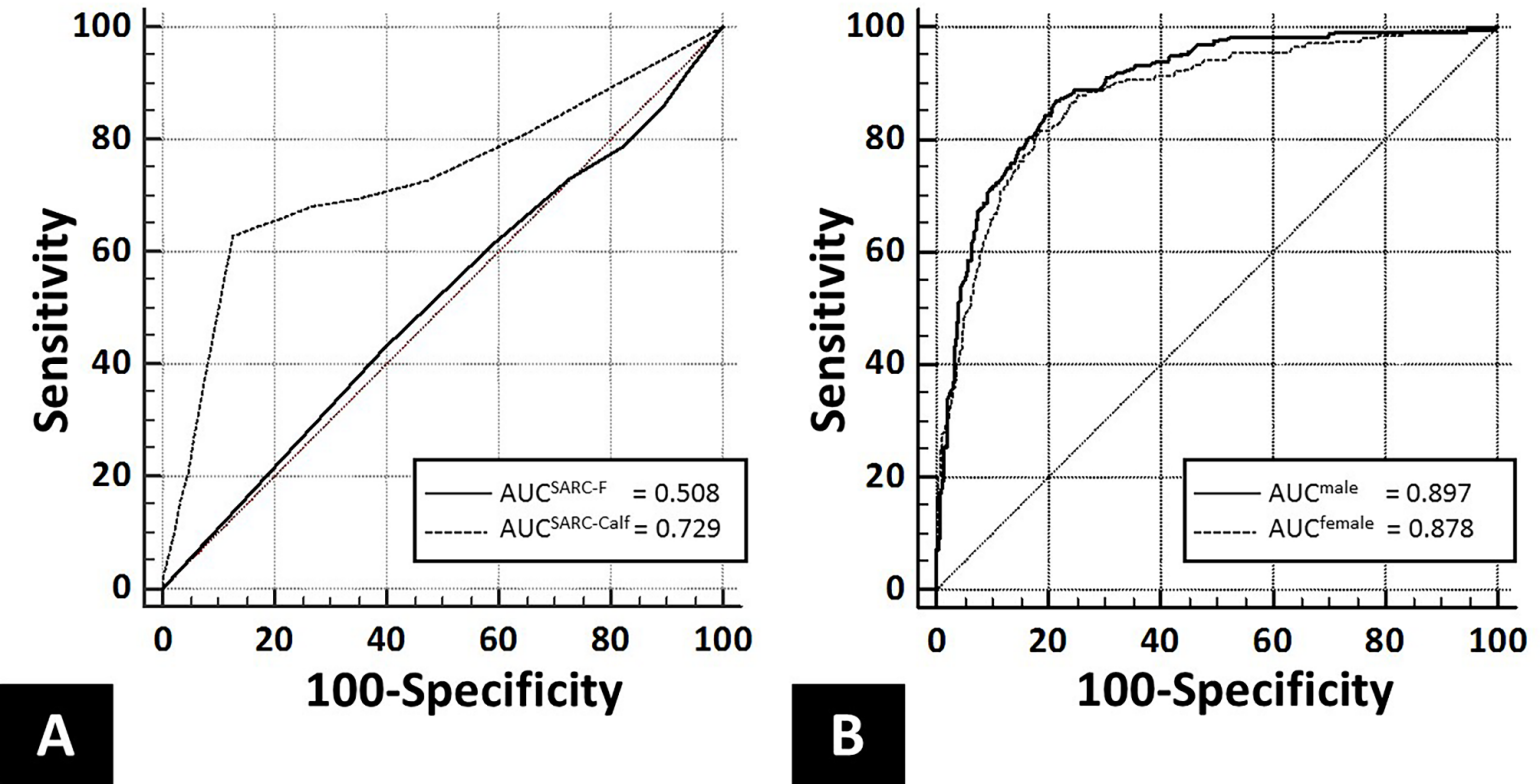
Receiver operating characteristic curves for diagnosing sarcopenia: **(A)** SARC-F and SARC-CalF questionnaires and **(B)** calf circumference measurements

Calf circumference demonstrated high sensitivity for diagnosing sarcopenia, with values of 93.3% (95% CI 89.3–96.1) in males and 91.4% (95% CI 86.7–94.8) in females. The specificity was moderate, reaching 63.1% (95% CI 59.3–66.8) in males and 59.4% (95% CI 56.7–62.0) in females. The AUC was 0.897 (95% CI: 0.874–0.920) for males and 0.878 (95% CI: 0.852–0.904) for females, suggesting high diagnostic accuracy and clinical utility (Fig. 3).

Compared with calf circumference, the SARC-F showed much lower sensitivity (21.1%, 95% CI 17.4–25.2) but higher specificity (82.5%, 95% CI 80.8–84.2). The SARC-CalF exhibited a low sensitivity of 38.7% (95% CI 34.1–43.4) and a high specificity of 92% (95% CI 90.8–93.2). The AUC values for the SARC-F and SARC-CalF were 0.508 (95% CI 0.477–0.538) and 0.729 (95% CI 0.699–0.759), respectively, indicating limited clinical utility (Table 3).

**Table 3:** Diagnostic performance of SARC-F, SARC-CalF, and calf circumference for diagnosing sarcopenia

Screening tests		AUC (95% CI)	Sensitivity % (95% CI)	Specificity % (95% CI)	PPV % (95% CI)	NPV % (95% CI)
SARC-F (≥ 4)		0.508 (0.477–0.538)	21.1 (17.4–25.2)	82.5 (80.8–84.2)	21.1 (17.9–24.7)	82.5 (81.7–83.2)
AWGS 2019 calf circumference	Male (< 34 cm)	0.897 (0.874–0.920)	93.3 (89.3–96.1)	63.1 (59.3–66.8)	47.7 (45.1–50.4)	96.3 (94.1–97.7)
	Female (< 33 cm)	0.878 (0.852–0.904)	91.4 (86.7–94.8)	59.4 (56.7–62.0)	25.7 (24.2–27.2)	97.8 (96.6–98.6)
SARC-CalF (≥ 11)		0.729 (0.699–0.759)	38.7 (34.1–43.4)	92 (90.8–93.2)	51.8 (47.1–56.5)	87.1 (86.3–88.0)
New cut-off calf circumference	Male (< 33 cm)	0.823 (0.791–0.855)	84.4 (79.1–88.8)	80.2 (76.9–83.2)	60.6 (56.7–64.4)	93.4 (91.3–95.0)
	Female (< 31 cm)	0.804 (0.770–0.839)	77.9 (71.6–83.3)	83 (80.9–85.0)	41.3 (38.0-44.7)	96.1 (95.0-96.9)
Revised SARC-CalF (≥ 11)		0.798 (0.774–0.822)	47 (42.3–51.7)	87.8 (86.3–89.2)	46.0 (42.3–49.9)	88.2 (87.3–89.1)

Abbreviations: AUC = area under the receiver operating characteristic curve; CI = confidence interval; NPV = negative predictive value; PPV = positive predictive value

### Impact of new cutoff scores on diagnostic performance for Sarcopenia

When the new cutoff scores defined by the maximum Youden index were applied for diagnosing sarcopenia (< 33 cm for males and < 31 cm for females), the sensitivity slightly decrease to 84.4% (95% CI 79.1–88.8) for males and 77.9% (95% CI 71.6–83.3) for females. However, the specificity improved, reaching 80.2% (95% CI 76.9–83.2) in males and 83% (95% CI 80.9–85.0) in females. Although the AUC values slightly decreased to 0.823 for males and 0.804 for females, they remained above 0.75, indicating clinical relevance. Furthermore, the newly established cutoffs demonstrated significantly improved overall diagnostic accuracy for detecting sarcopenia compared to the criteria outlined in the AWGS 2019 guidelines (Table 4).

**Table 4:** Comparison of diagnostic performance between AWGS 2019 and new calf circumference cutoff values for sarcopenia diagnosis

Diagnostic performance	AWGS 2019 cutoff value (< 34 cm for males and < 33 cm for females)	New cutoff value (< 33 cm for males and < 31 cm for females)	*p*-value
AUC [SE] (male) AUC [SE] (female) Sensitivity	0.897 [0.012] 0.878 [0.013] 92.4% (89.5-94.7%)	0.823 [0.012] 0.804 [0.013] 81.4% (77.4-84.9%)	< 0.001* < 0.001* < 0.001**
Specificity	60.6% (58.4-62.7%)	82.1% (80.3-83.7%)	< 0.001**
Accuracy	66.4% (64.5-68.2%)	82.0% (80.4-83.5%)	< 0.001**

* DeLong’s test for AUC comparisons

** McNemar’s test for other diagnostic performance comparisons

Abbreviations: AUC = area under the receiver operating characteristic curve; SE = standard error

## Discussion

Sarcopenia is characterized by the progressive loss of skeletal muscle mass with age. It is a critical concern because of its strong associations with reduced physical function, increased risk of falls, and a higher incidence of fractures, especially among the elderly population [28].

Our study revealed that the prevalence of sarcopenia among community-dwelling older adults in Thailand is 18.1%, which is relatively high compared with the 10–16% prevalence reported in elderly populations from other countries [29]. This discrepancy may result from differences in diagnostic methods and variations in the demographic groups studied. Given the associated risks, effective screening for sarcopenia is essential to prevent related complications. However, our findings suggest that the SARC-F and SARC-CalF questionnaires are not sufficiently effective for detecting sarcopenia in elderly individuals. In contrast, calf circumference measurement alone appears to be a more reliable indicator for this purpose.

While the SARC-F and SARC-CalF questionnaires demonstrated high sensitivity as tools for identifying possible sarcopenia, their sensitivity for confirming actual sarcopenia was much lower. This outcome aligns with our finding that 93.7% of individuals classified as having possible sarcopenia were confirmed to have the condition, whereas only 19.3% had sarcopenia (Fig. 1). These findings suggest that the SARC-F and SARC-CalF questionnaires are useful for initial screening in primary healthcare or community preventive settings. However, further evaluation with tools such as a hand dynamometer or physical performance tests may still be necessary for individuals with abnormal scores. Nevertheless, conducting additional tests can be challenging in settings lacking the required equipment or trained personnel, potentially leading to unnecessary referrals for muscle mass measurement.

Our findings are consistent with those of previous studies showing that the SARC-F has low sensitivity but high specificity for diagnosing sarcopenia [30, 31]. This characteristic may be because the questionnaire’s items are more relevant to elderly individuals who are severely frail than to those who are relatively strong. Additionally, none of the questions directly assess muscle mass, a critical criterion in the diagnosis of sarcopenia.

Our study suggests that calf circumference may be a more effective tool than the SARC-F and SARC-CalF questionnaires for both screening and diagnosing sarcopenia. This conclusion is supported by its AUC value being greater than 0.75, which is considered clinically significant. Calf circumference, as an anthropometric measure, reflects factors such as nutritional status, body mass index, and muscle mass, all of which are recognized risk factors for the development of sarcopenia [15, 32, 33].

A previous study demonstrated a strong correlation between calf circumference and calf muscle mass in elderly individuals, with correlation coefficients of *r* = 0.908 in males and *r* = 0.892 in females, both with *p*-values of < 0.05 [34]. ASM, which includes calf muscle mass, is a key diagnostic criterion for sarcopenia. Adjusting the cutoff point for calf circumference to < 31 cm for females and < 33 cm for males increased the specificity from 60.6 to 82.1%, with only a slight decrease in sensitivity from 92.4 to 81.4%. However, the overall accuracy improved from 66.4 to 82%. This approach may be particularly suitable for screening for sarcopenia, especially in settings where advanced diagnostic tools such as BIA or dual-energy X-ray absorptiometry are not readily available. Additionally, since sarcopenia is not an immediately life-threatening condition, it may be acceptable if some cases are missed during the screening process [35].

To improve the accuracy of the SARC-F questionnaire, incorporating additional parameters that reflect muscle quantity may be necessary. For example, adding factors such as age, arm circumference, and body mass index—parameters shown in previous studies to enhance screening accuracy—could be beneficial [36, 37]. This approach aligns with our findings that including calf circumference improves the sensitivity and specificity of sarcopenia screening. However, for community-level screening, measuring calf circumference remains a practical option. It is a simple, noninvasive, and inexpensive tool that can be used in various settings. Additionally, studies have demonstrated a positive correlation between calf circumference and muscle mass measured by both BIA and dual-energy X-ray absorptiometry, regardless of factors such as obesity and age [38]. Therefore, calf circumference measurement is likely the most suitable method for large-scale population screening.

The strength of this study lies in its distinction as the first large-scale national investigation of sarcopenia in Thailand. The sample included older adults aged 60 years and above from community settings, and it encompassed all six geographically diverse regions of the country. Moreover, this study is the first to show that the SARC-F and SARC-CalF screening questionnaires are not clinically effective. In contrast, we identified calf circumference as the most effective method for sarcopenia screening. Highlighting calf circumference as a reliable and cost-effective tool is particularly beneficial for large-scale or resource-limited settings. This approach has led to the establishment of a new national cutoff point for low calf circumference, specifically tailored to Thai older adults.

However, there are limitations to consider. The measurement of calf circumference may not fully account for the influence of adipose tissue and limb edema, which could affect the accuracy of the assessment. Additionally, the use of BIA instead of dual-energy X-ray absorptiometry may have introduced variability due to differences in participant hydration levels, potentially impacting the reliability of muscle mass measurements.

## Conclusions

The prevalence of sarcopenia in Thailand is relatively high. The SARC-F questionnaire has proven ineffective for diagnosing sarcopenia, whereas calf circumference has been shown to be effective as a screening tool. The incorporation of additional parameters into the SARC-F questionnaire may enhance its diagnostic accuracy, but further research is needed to validate this approach.

## Data Availability

The data supporting this study’s findings are available from the corresponding author upon reasonable request. The data are not publicly available due to privacy or ethical restrictions.

## Abbreviations

AWGS: Asian Working Group for Sarcopenia
ASM: Appendicular skeletal muscle mass
5TSTS: Five-time sit-to-stand
BIA: Bioelectrical impedance analysis
AUC: Area under the receiver operating characteristic curve
